# Psychometric properties of the Pittsburgh Fatigability Scale for assessing physical and mental fatigability in Brazilian older adults (PFS-Brasil)

**DOI:** 10.1590/1414-431X2025e14619

**Published:** 2026-02-16

**Authors:** M.S.B. Santos, J. Bento-Torres, L.L. Santana, A.A. da Costa, I.P.P.C. Gesta, L.M. Silva, C.W. Picanço-Diniz, N.W. Glynn, N.V.O. Bento-Torres

**Affiliations:** 1 Programa de Pós-Graduação em Ciências do Movimento Humano, Laboratório de Investigações em Neurodegeneração e Infecção, Universidade Federal do Pará, Belém, PA, Brasil; 2 Programa de Pós-Graduação em Ciências do Movimento Humano, Universidade Federal do Pará, Belém, PA, Brasil; 3 Laboratório de Investigações em Neurodegeneração e Infecção, Faculdade de Fisioterapia e Terapia Ocupacional, Universidade Federal do Pará, Belém, Pará, Brasil; 4 Programa de Pós-Graduação em Neurociência e Biologia Celular, Laboratório de Investigações em Neurodegeneração e Infecção, Universidade Federal do Pará, Belém, Pará, Brasil; 5 Department of Epidemiology, School of Public Health, University of Pittsburgh, Pittsburgh, PA, USA

**Keywords:** Fatigue, Older adults, Psychometric properties, Perceived fatigability, Quality of life, Assessment

## Abstract

Fatigability is critical for understanding older adults' physical and mental health. The Pittsburgh Fatigability Scale (PFS) is widely employed to measure perceived fatigability, reflecting how fatigue impacts performance and its association with adverse health outcomes. This study aimed to evaluate the psychometric properties of the PFS adapted for Brazilian older adults (PFS-Brasil), focusing on perceived physical and mental fatigability. Confirmatory factor analysis (CFA) validated the bifactorial model. Internal consistency was assessed using Cronbach's alpha, while test-retest reliability was evaluated through the intraclass correlation coefficient (ICC) and Bland-Altman Plot. Convergent validity was determined by correlating PFS-Brasil score with physical and cognitive performance measures, and ceiling and floor effects were analyzed. CFA confirmed the two-factor structure of the PFS-Brasil. The Physical and Mental subscales showed high internal consistency (α=0.89 and 0.86, respectively). Test-retest reliability demonstrated good agreement (ICC for Physical=0.84; Mental=0.83). Higher fatigability score correlated with poorer physical performance on the Short Physical Performance Battery (SPPB) and the 6-Minute Walk Test (6MWT), with physical score showing weak to moderate correlations (P=-0.22 to -0.37) and mental score showing weak correlations (P=-0.20 to -0.25). Mental fatigability was weakly correlated with inhibitory control. The PFS-Brasil demonstrated robust psychometric properties, supporting its reliability and validity for assessing perceived physical and mental fatigue in older adults. Its use is recommended in clinical and research settings to identify individuals at risk of physical and psychological decline, promoting better health outcomes and quality of life in aging.

## Introduction

Perceived fatigability is a psychophysiological condition characterized by an increased subjective experience of physical or mental fatigue following activities of standardized intensity and duration. This construct provides a sensitive measure of how fatigue limits an individual's performance (1,2). Physical fatigability is highly prevalent among older adults, often more so than mental fatigability. Studies have reported prevalence rates for physical fatigability ranging from 19.4 to 89.5%, while mental fatigability ranges from 14.5 to 67.2% (3).

Fatigability increases with age, becoming more pronounced among nonagenarians and centenarians (3,4). The relationship between fatigability and aging outcomes is complex, affecting physical and cognitive domains (5,6). Greater fatigability has been associated with reduced physical activity, worse physical function and performance, impaired cognitive function, slower gait speed, higher body mass index (BMI), increased levels of inflammatory markers, cardiovascular risk, poor fitness, depressive symptoms, and poor sleep quality as measured by actigraphy (3). Fatigability also predicts faster declines in physical function, increased functional limitations, mobility impairments, cognitive decline, fall risk, and mortality (7,8).

Given these associations, fatigability is an early marker of functional decline, often preceding declines observed in physical function assessments used in clinical or research settings (2). Additionally, fatigability has been proposed as a prognostic indicator of phenotypic aging and an early predictor of adverse outcomes in older adults (2). Greater fatigability can lead to activity limitations and participation restrictions, especially in individuals with reduced physical fitness or chronic conditions, ultimately affecting their functionality and quality of life (9,10).

The Pittsburgh Fatigability Scale (PFS) is the only validated patient-reported instrument designed specifically to assess older adults' perceived physical and mental limitations caused by fatigue (11,12). The PFS measures perceived fatigability in relation to standardized tasks of specific intensity and duration, capturing how fatigue affects older adults' involvement in physical activities, social activities, and activities of daily living (11).

Although Brazilian Portuguese adaptations of generic fatigue instruments are available, these tools quantify overall fatigue intensity or impact without referencing task-specific demands or differentiating physical from mental fatigability. Consequently, they are less sensitive to early functional decline in community-dwelling older adults, providing limited guidance for personalizing activity-based interventions. The PFS addresses this limitation by linking fatigue perceptions to specific activities and by offering a separate subscale for physical and mental fatigue. Initially developed in English, the PFS has undergone translation and validation in various languages and populations (13,14). These efforts facilitate the comparison of perceived fatigability across different populations and help bridge the gap between research and clinical practice, providing a cost-effective tool to support evidence-based interventions.

Despite the broad application of the PFS, a validated version for Brazilian Portuguese (PFS-Brasil) has not yet been established (12,15-19). Adequate cross-cultural adaptation is essential to ensure the reliability and validity of an instrument across diverse contexts (13,14), and is particularly relevant in Brazil, where socioeconomic diversity, linguistic nuances, and lifestyle patterns may influence self-reported fatigue. Accordingly, this study had two aims: i) to translate and cross-culturally adapt the original PFS for use in Brazil and ii) to conduct a comprehensive psychometric evaluation of the resulting PFS-Brasil - including analyses of factorial validity, internal consistency, test-retest reliability, and convergent validity - to confirm its suitability for assessing perceived physical and mental fatigability in community-dwelling Brazilian older adults.

Incorporating the PFS-Brasil into routine geriatric assessments within the primary care setting could facilitate early identification of older adults at risk of functional decline. Its task-anchored score enable exercise professionals to individualize exercise prescriptions, rehabilitation goals, and multidisciplinary care plans, thereby optimizing patient outcomes and resource allocation. At the population level, widespread use of the PFS-Brasil can support national healthy-ageing initiatives to promote healthy aging by mapping regional profiles of perceived fatigability, guiding investment in community-based physical-activity and rehabilitation programs, and ultimately reducing disability-related expenditures.

## Material and Methods

### Study design and participant selection

We performed a study to assess the psychometric properties of the PFS-Brasil with permission of the developer and the University of Pittsburgh. The PFS-Brasil adaptations followed the translation-back translation methodology (13). This study was approved by the Health Science Institute of the Federal University of Pará Review Board (Approval Number: 5.325.956). Informed written consent was obtained before data collection, and all procedures adhered to the Declaration of Helsinki. Participants received a copy of their personal assessment results, a detailed explanation of their performance, and a health-related professional orientation by a certified physical therapist.

Sample size was determined according to the widely accepted criterion of at least 10 participants per item when conducting psychometric analysis (14). Given that the PFS-Brasil is a ten-item scale, a minimum of 100 participants were required (10 items × 10 participants/item). Data were collected at the Neurodegeneration and Infection Research Laboratory at the University Hospital João de Barros Barreto, Belém (PA), Brazil, from October 2022 to August 2023.

Community-dwelling older adults were invited to participate through advertisements on social media, seniors' centers, university outreach programs, university surroundings, and word-of-mouth. Eligible participants were required to be 60 years or older, have Brazilian Portuguese as a first language, have normal or corrected-to-normal visual acuity, and have preserved mobility (self-reported ability to walk 400 m at a usual pace). Exclusion criteria included a history of head trauma with loss of consciousness, inability to communicate, any orthopedic, visual, or hearing condition that limits test performance, major depression symptoms based on the DSM-5 criteria, self-reported neurological or neuromuscular disease, and undergoing cancer treatment.

Questionnaire data, including demographics, anthropometrics, and health-related information, were collected during individual interviews. The PFS-Brasil was self-administered, followed by cognitive and physical assessments. Each assessment session lasted approximately 60 min. Cognitive tests were always administered before the physical tests to prevent potential carry-over effects of physical activity on cognitive performance. A 5-min mandatory rest period was scheduled at the session midpoint, and additional breaks were provided whenever requested by the participant to minimize potential fatigue. After 30 days, participants were reassessed with the PFS-Brasil to evaluate test-retest reliability.

### The Pittsburgh Fatigability Scale - Brazilian Portuguese version

The PFS is a validated 10-item self-administered scale (11,12) designed to assess physical and mental fatigability anchored to physical, social, and daily living activities of defined intensities and duration. Participants reported how they feel or expect to feel immediately after performing ten different activities on a six-point scale, ranging from 0 (no fatigue) to 5 (extreme fatigue). The scale assesses physical and mental fatigue in separate subscales, with the total score calculated by summing the ten items up to 50 points each. Higher PFS scores indicate greater fatigability. More severe physical fatigability was defined as ≥15 points, and more severe mental fatigability as ≥13 points (8,11,12).

We performed a translation/back-translation process for cross-cultural adaptation of the PFS-Brasil (13). Briefly, the six-step protocol performed included: 1) translation by two independent native Portuguese speakers of the PFS from English to Brazilian Portuguese (T1 & T2); 2) synthesis of T1 and T2 into one version (T12), and a pilot study with 15 older adult participants to gather feedback on the T12; 3) back-translation of the T12 version by two bilingual translators, native English speakers fluent in Brazilian Portuguese (BT1 & BT2). The back-translated versions (BT1 and BT2) were synthesized into one (BT12); 4) the documents were reviewed by an expert committee comprising a physical therapist (PhD), an occupational therapist (MSc), a physician (PhD), and an exercise physiologist (PhD). The committee members included clinical professionals who work with older adults and researchers in the field of aging; 5) to test the PFS-Brasil, 50 participants completed the scale and were interviewed to ensure clarity and comprehension of each questionnaire item and response; 6) the preliminary and final documentation was submitted to the developers for appraisal of the adaptation process and suggestions throughout the process.

The PFS-Brasil's convergence and construct validity were assessed through tests of physical performance, level of physical activity, functional capacity, and cognitive performance.

### Physical performance and cognitive measures

Physical performance was assessed using the Short Physical Performance Battery (SPPB) test, which predicts mobility problems, hospitalizations, and physical disability in older adults (20). This battery ranges from 0 (worst performance) to 12 points (best performance), and includes static balance, gait speed, and lower limb strength tests. Scores from 0 to 3 indicate very poor performance, 4 to 6 low performance, 7 to 9 moderate performance, and 10 to 12 good performance.

The level of physical activity was assessed using the International Physical Activity Questionnaire (IPAQ, long version, usual week). IPAQ analyzes metabolic equivalents of task (MET) per week on walking and moderate and vigorous physical activity across different domains of physical activity (work, transport, domestic activities, and leisure time). Only activities with at least ten continuous minutes were considered (21).

Functional exercise capacity was measured by the 6MWT as an indirect measure of cardiorespiratory fitness. Participants were instructed to walk at their own pace on a flat surface for 6 min, with the total distance covered in meters used as the performance measure. Two trials were performed with a five-minute interval between each, with the greatest distance used for data analysis (22).

Cognitive performance was assessed using the Mini-Mental State Examination (MMSE), the Trail Making Test, and the Flanker Test. The MMSE (0-30 points) is a screening test for cognitive impairment, with the cutoff score adjusted for educational levels: illiterate, 13 points; 1-7 years of schooling, 18 points; >7 years of education, 26 points (23). The Trail Making Test screens for neurological diseases and neuropsychological impairment. It comprises two parts (Trail A and Trail B), which require visual search, motor speed skills, and mental flexibility. A 300-s cutoff time was applied, with errors not directly contributing to the final score (24). In Trail A, participants link 25 numbered circles in ascending order. In Trail B, participants link numbers and letters in ascending order (e.g., 1-A, 2-B). The Flanker Test, an indicator of executive control and selective attention, was performed using the Psychology Experiment Building Language (PEBL) software. It included 144 trials with congruent and incongruent Flanker conditions presented randomly. Outcome measures included response time and the number and proportion of correct answers.

### Statistical analyses

Demographic data (age, sex, race, and education) and PFS-Brasil Physical Mental scores were analyzed using descriptive statistics, including mean, standard deviation, and/or frequency measurements (%). Normality distribution was examined using the Kolmogorov-Smirnov test with Lilliefors correction. Data were analyzed using the Statistical Package for Social Sciences (SPSS, 25.0, IBM, USA) and JASP software (0.17.1).

Confirmatory factor analysis was used to test the bifactorial model (12,17). Data were analyzed using the R programming language through the JASP software interface (v. 0.17.1). The Lavaan package (Latent Variable Analysis) (25) was used to perform confirmatory factor analysis based on the weighted least squares mean and variance-adjusted estimator, a robust estimator suitable for categorical data (26). This technique analyzes a factorial structure underlying a measurement, and its results are assessed based on adjustment indicators, which describe a measurement of the model that presents convergence or divergences from the model found in real data. The following adjustment indexes were used: a) χ^2^, in which non-significant values indicate ideal adjustment of the model; b) the ratio χ^2^/df, which presents adequate results when ≤5; c) comparative fit index (CFI≥0.95); d) Tucker-Lewis Index (TLI), presenting adequate model fit results when ≥0.90, and excellent when ≥0.95; and e) standardized root mean square residual, presenting satisfactory model fit when ≤0.08 and excellent model fit when below 0.06 (27).

The internal consistency of the PFS-Brasil was assessed using Cronbach's alpha (α), with values above 0.70 indicating satisfactory internal consistency. The test-retest reliability was calculated with an intraclass correlation coefficient (ICC) for all participants who attended the recall to respond a second time to the PFS-Brasil. A two-way mixed effects model was applied. ICC≥0.70 indicated satisfactory reliability between the two time points (28). Seven participants did not return for the 30-day retest. These individuals were excluded list-wise from the test-retest reliability analyses but were retained in all other analyses that did not require retesting.

Agreement was assessed using the Bland-Altman plot, which describes the agreement between two quantitative measurements (test and retest) by constructing limits based on the mean and standard deviation of the differences between the two measurements, assessing the repeatability of the measures of the two-time points (29). A scatterplot was generated, with the difference between test and retest score plotted on the Y-axis and PFS-Brasil Physical and Mental score plotted on the X-axis. Limits were defined within a 95% confidence interval (mean of differences ± 1.96 × standard deviation of differences) (29).

Measurement error was calculated using the standard error of measurement (SEM), which measures the precision of the sample mean (30). Good reliability reference values were considered ≤10% (16). The smallest detectable change (SDC) was calculated as SDC=1.96 × √^2^ × SEM (31). Both absolute SDC and the percentage of the scale range (0-50 points) were calculated.

Convergent validity (the degree to which theoretically related measures are related) was assessed by correlating PFS-Brasil Physical and Mental subscale score with physical performance, physical activity, and cognitive performance measures. Due to the non-normal data distribution, Spearman's correlation coefficient was used, with P values of 0.10-0.39 indicating weak correlation, 0.40-0.69 moderate correlation, 0.70-0.89 strong correlation, and ≥0.90 very strong correlation (32).

The presence of ceiling and floor effects was analyzed by determining the percentage of participants with maximum (50 points) and minimum (0 points) scores. Ceiling and floor effects were considered present when ≥20% of participants scored 0 (no fatigue) or 5 (extreme fatigue) on all ten items, with a minimum acceptable criterion of ≤20% (16). Results are reported as percentages.

## Results

### Participant characteristics

One hundred and twenty-one community-dwelling older adults participated in this study, with a mean age of 70.3±6.4 years and an average education level of 12±4.7 years. The majority of participants were women (86%). Most participants were either single or divorced (37.2%), identified as from a multiracial origin (68.6%), and were classified as overweight or obese. Detailed demographics, clinical characteristics, and the PFS-Brasil Physical and Mental scores for the sample are presented in Table 1.

**Table 1:** Participant characteristics.

Characteristics	Validation sample (n=121)
Age (years)	70.3±6.4
Female	104 (86.0)
Marital status	
Married	40 (33.1)
Single/Divorced	45 (37.2)
Widow	36 (29.7)
Education	12±4.7
1-4 years of education	12 (10.0)
5-8 years of education	17 (14.0)
9-12 years of education	28 (23.1)
13-16 years of education	42 (34.7)
≥17 years of education	22 (18.2)
Racial groups	
White	20 (16.5)
Black	16 (13.2)
Multiracial	83 (68.6)
Other	2 (1.7)
Body mass index (BMI, kg/m^2^)	29.2±4.9
Eutrophy	28 (23.1)
Overweight	44 (36.4)
Obesity	49 (40.5)
Fatigability score	
PFS-Brasil Physical score	15.0±9.3
PFS-Brasil Mental score	7.8±7.7
More severe physical fatigability*	65 (53.7)
More severe mental fatigability*	33 (27.3)

Data are reported as means±SD or n (%). *More severe physical fatigability was defined as ≥15 points on the PFS, and greater mental fatigability was defined as ≥13 points on the PFS. PFS: Pittsburgh Fatigability Scale.

### Translation process

During the translation process, only minor modifications were necessary. Kilograms (kg) replaced pounds (lbs) to align with the national unit system. Terms such as “baking”, “senior center”, “shoveling snow”, “bridge”, “hiking”, and “host” were replaced with energy expenditure equivalent activities more common among older adults in Brazil or were omitted. Based on suggestions and difficulties noted by the expert committee and pilot study participants, definitions for mental fatigue and vigorous and moderate physical activities were formulated. These definitions are intended exclusively for staff and researchers to clarify concepts if participants request, ensuring standardized staff interaction.

The documentation was submitted to the developers for appraisal of the adaptation process. Dr. Glynn reviewed the pre-final version of the PFS-Brasil and had it evaluated by two external, independent bilingual consultants. The suggestions received were discussed in a meeting to address the final version of the PFS-Brasil. Fifty participants completed the test of the final version, providing feedback on language clarity and comprehension. They also responded to an open-ended question for suggestions. Any item reported as unclear by 20% or more participants would have been re-evaluated, but no revisions were necessary. The PFS-Brasil is free of charge for academic purposes upon request to Dr. Nancy Glynn (https://www.sph.pitt.edu/pittsburgh-fatigability-scale).

### Scale bifactorial model - confirmatory factor analysis

The confirmatory factor analysis indicated a satisfactory fit of the bifactorial model of the PFS-Brasil Physical and Mental subscale. Factor 1 consisted of items a, b, c, d, g, and j, which included physical activities of varying intensities: light (walking and daily home activities), moderate (strength training), and high intensities (walking, gardening, outdoor activities, sports activities, and physical exercise). Factor 2 comprised items e, f, h, and i, which pertained to social participation activities and sedentary behavior. All factor loadings were significantly different from zero (λ≠0; t>1.96 P<0.05) (Table 2).

**Table 2:** Factor loading of the Pittsburgh Fatigability Scale - Brasil version (PFS-Brasil) (n=103).

Items	Item description	Physical Fatigability Subscale		Mental Fatigability Subscale	
		Factor 1	Factor 2	Factor 1	Factor 2
A	Leisurely walk for 30 min	0.742		0.683	
B	Brisk or fast walking for 1 h	0.700		0.785	
C	Light household activity for 1 h	0.693		0.851	
D	Heavy gardening or outdoor work for 1 h	0.775		0.777	
G	Moderate- to high-intensity strength training for 30 min	0.795		0.793	
J	High-intensity activity for 30 min	0.746		0.799	
E	Watching TV for 2 h		0.298		0.625
F	Sitting quietly for 1 h		0.350		0.575
H	Participating in a social activity for 1 h		0.797		0.783
I	Hosting a social event for 1 h		0.836		0.688

The results demonstrated an excellent fit for the model, with the Physical subscale showing CFI=0.95, TLI=0.93, and RMSEA=0.07, and the Mental subscale showing CFI=1.00, TLI=0.99, and RMSEA=0.02 (Table 3).

**Table 3:** Model fit results from confirmatory factor analysis in the Pittsburgh Fatigability Scale - Brasil version (PFS-Brasil) (n=103).

	χ^2^ (df)	χ^2^/df	CFI	TLI	RMSEA
Physical Fatigability Subscale	48.55 (34)	1.42	0.95	0.93	0.07
Mental Fatigability Subscale	35.06 (34)	1.03	1.00	0.99	0.02

χ^2^: chi-squared; χ^2^/df: chi-squared ratio by degree of freedom; CFI: comparative fit index; TLI: Tucker-Lewis Index; RMSEA: standardized root mean square residual.

### Reliability

The reliability, internal consistency, measurement error, and agreement indices are summarized in Supplementary Table S1. The internal consistency analysis resulted in satisfactory Cronbach's alpha values of 0.80 and 0.78 for the PFS-Brasil Physical and Mental subscale, respectively. One hundred and fourteen participants completed the PFS-Brasil a second time, and their data were included in the test-retest analysis. These data were collected within 30 days of the first assessment (27.1±5.9 days between assessments). Test-retest reliability also indicated excellent internal consistency, with ICC values of 0.84 (95%CI: 0.80-0.88) for the PFS-Brasil Physical and 0.83 (95%CI: 0.78-0.87) for the Mental subscale, respectively.

**Figure 1:**
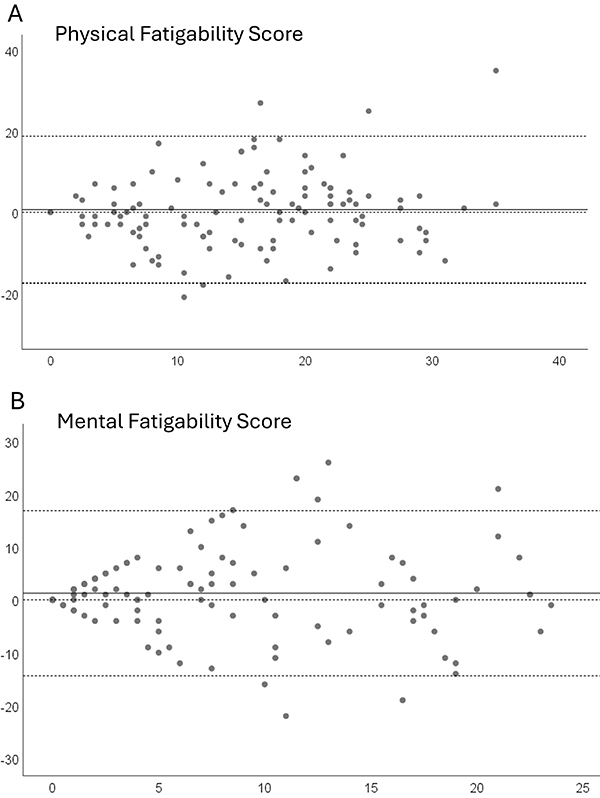
Bland-Altman Plot for the Pittsburgh Fatigability Scale - Brasil version. Physical Fatigability Score (**A**) and the Mental Fatigability Score (**B**).

Within the limits of sampling variation, the agreement measures for the SDC and SEM demonstrated good levels. The SDC was 10 points (20%) for the Physical subscale and 8 points (17%) for the Mental subscale. The SEM was 7% for the Physical subscale and 6% for the Mental subscale. These values suggest minimal differences between the instrument means over the weeks, indicating good interobserver reliability and a low probability of random and systematic error. The Bland-Altman plots showed good agreement for both PFS-Brasil subscales, with a mean difference of 0.64 (95%CI: -17.53 to 18.80) for the Physical score and a mean difference of 1.26 (95%CI: -14.38 to 16.89) for the Mental score (Figure 1).

### Convergent validity

The PFS-Brasil Physical and Mental scores exhibited a weak inverse correlation with the total SPPB score, gait speed, and 5-times chair stand components (Table 4). These findings indicated that greater fatigability was associated with worse performance on these tests. Additionally, lower performance, as measured by shorter distances walked on the 6MWT, showed a weak correlation with higher PFS-Brasil Physical score and a weak correlation with higher PFS-Brasil Mental score.

**Table 4:** Convergent validity. Correlation coefficients for Pittsburgh Fatigability Scale - Brasil version (PFS-Brasil) physical score and mental score with physical and cognitive performance.

Instruments	Spearman Correlation		
	Mean±SD	Physical subscale score	Mental subscale score
Short Physical Performance Battery (SPPB) (Total score, 0-12)	11.1±1.1	-0.22*	-0.20**
Gait speed (m/s)	1.40±0.5	-0.35***	-0.21*
5-chair stand (s)	11.3±3.4	0.21**	0.30***
Balance (points)	3.9±0.3	-0.13	-0.08
Six-minute walk test (meters)	467.3±88.5	-0.37***	-0.25**
Body mass index (BMI)	29.1±4.9	0.19**	0.13
Physical Activity (IPAQ) (MET-min/week)			
Walking	536.9±1.244.3	-0.10	-0.002
Moderate physical activity	2569.4±12.062.6	-0.10	-0.10
Vigorous physical activity	517.4±767.9	-0.09	-0.14
Total physical activity	5111.0±13168.2	-0.19*	-0.13
Trail Making Test			
Trail A (s)	79.8±42.5	0.13	0.12
Trail B (s)	193.7±85.4	0.02	-0.03
Flanker Test			
Response Time - Correct responses - Congruent condition (ms)	575.4±74.3	0.08	0.06
Response Time - Correct responses - Incongruent condition (ms)	598.71±85.4	-0.02	0.01
Total Correct - Congruent condition (n)	53.79±24.5	-0.14	-0.18*
Total Correct - Incongruent condition (n)	51.04±24.6	-0.14	-0.20*
Conflict Cost	27.07±83.9	-0.08	-0.80
Mini-Mental State Examination (0-30 points)	27.9±2.2	-0.12	0.01

Physical score (0-50 points). Mental score (0-50). IPAQ: International Physical Activity Questionnaire. *P≤0.05; **P≤0.01; ***P≤0.001.

The PFS-Brasil Mental score demonstrated a weak inverse correlation with the total number of correct answers in the congruent and incongruent conditions of the Flanker Test, indicating that greater mental fatigability was associated with lower performance on the attention-demanding tasks (Table 4).

Moreover, a higher PFS-Brasil Physical score was also correlated with significant risk factors for poor health status in aging, as measured by BMI and physical activity levels (Table 4). Specifically, there was a weak correlation between a higher PFS-Brasil Physical score and higher BMI and a weak correlation between a higher PFS-Brasil Physical score and lower total physical activity as assessed by the IPAQ. PFS-Brasil Physical and Mental subscale scores were correlated (P=0.69, P<0.01).

## Discussion

Our findings showed that the PFS-Brasil exhibited robust psychometric properties for assessing perceived physical and mental fatigability in community-dwelling older adults. The instrument demonstrated satisfactory construct validity, high internal consistency, test-retest reliability, and strong agreement across assessments. These results confirmed that the PFS-Brasil is a valid and reliable tool for evaluating fatigability in older Brazilian adults. Its simplicity makes it well suited for academic research and clinical practice.

The translation of the PFS into Brazilian Portuguese followed established guidelines for the translation and adaptation of outcome measurement instruments, ensuring linguistic and cultural relevance without compromising the validity of the original instrument (13,33). The expert committee, in collaboration with the instrument's developer, made minor adjustments to the PFS-Brasil to enhance item clarity and ensure cultural relevance. Confirmatory factor analysis confirmed good construct validity and supported the suitability of the bifactorial model, with distinct Physical and Mental subscales. These findings align with previous studies (12,17,18) and adhere to recommendations regarding the minimum number of items required per factor for accurately measuring latent variables. Our results revealed that Factor 1 encompasses physical activities across light, moderate, and high intensities, while Factor 2 includes social participation and sedentary behaviors. Our findings demonstrated that there was no cross-loading between the two factors, underscoring the clear distinction between the constructs they represent. The PFS-Brasil Physical (Cronbach's α=0.80) and Mental (Cronbach's α=0.78) subscales showed strong internal consistency and agreement. Both subscales also exhibited convergent validity with measures of physical performance, including the SPPB, gait speed, five-chair stand test, 6MWT, and physical activity levels, as well as cognitive performance assessed by the Flanker Test.

Higher PFS-Brasil score, reflecting greater physical and mental fatigability, were weakly associated with poorer physical performance. These correlations indicated that the PFS-Brasil effectively captured the physical and psychological fatigability experienced by older adults, even within a highly functional population, such as the participants in this study. The scale's association with physical performance measures, particularly the SPPB, highlighted its potential utility in clinical settings where monitoring physical function is essential for assessing health outcomes and guiding interventions (34).

The PFS-Brasil showed weak correlations with the number of correct responses on the inhibitory control assessment test. Similarly, previous validation studies reported very weak correlations, ranging from 0.04 to 0.17, highlighting the modest relationship between fatigability and inhibitory control (12,17). Some studies found no significant correlations between the PFS and cognitive performance measures or task-based assessments in healthy older adults, further suggesting that fatigability may not strongly relate to cognitive performance in this population (15,18). Although the PFS Mental subscale correlated with other self-reported fatigue measures, it did not align with task-based fatigability assessments. This discrepancy may stem from overlapping terminology and participants' challenges in differentiating between physical and cognitive fatigability within the context of the PFS (15). Although the PFS items initially seem to focus primarily on physical activities, many of these activities also demand mental effort for effective planning and execution (12). Given the limited understanding of the underlying concepts and mechanisms - mainly how physical activities can also induce mental fatigue - it is plausible that some older adults did not associate the tasks in the PFS with mental fatigue. Moreover, functional illiteracy remains common among Brazilian older adults, and low health-literacy levels can impair the comprehension of abstract constructs such as “mental fatigue”, potentially reducing the sensitivity of a cognitively demanding task like the Flanker Test (35,36). Future studies should consider alternative cognitive measures that place fewer reading or executive-instruction demands, or stratify analyses by validated health-literacy levels, to better represent the mental fatigability component in this population.

The internal consistency analysis of the PFS-Brasil was consistent with the original version and validations in other languages. The Cronbach's alpha values for the Physical subscale were comparable to those reported in previous studies, ranging from 0.83 to 0.88. For the Mental subscale, the PFS-Brasil's alpha value was slightly below the range observed in other translations, which reported values between 0.80 and 0.92 (11,12,17-19,37). These findings indicated that the scale's items are highly correlated, ensuring a reliable measure of perceived fatigability. The consistent results from previous translation studies further highlight the strong psychometric properties of the PFS, demonstrating the reliability and validity of both its Physical and Mental subscales across various languages, including Brazilian Portuguese.

Reliability assesses the reproducibility of an instrument by evaluating its ability to produce stable and reliable scores over time. The ICC values for the Physical subscale (0.84; 95%CI: 0.80-0.88) and Mental subscale (0.83; 95%CI: 0.78-0.87) indicated good agreement between test and retest, confirming that the PFS-Brasil consistently measured fatigability over time. In this study, the test-retest interval was approximately 30 days, aligning with similar validation studies conducted with the same population (11,12). The reliability results were further supported by the SEM, the SDC, and the Bland-Altman plot, which demonstrated homoscedasticity across both the Physical and Mental subscales of the PFS-Brasil. These findings align with those from the Dutch version of the PFS, the only other validation study to analyze agreement data, reinforcing the consistency and reliability of the PFS-Brasil (17). These findings suggested that the PFS-Brasil is a reliable and stable instrument for accurately assessing physical and mental fatigability in older Brazilian adults, with performance comparable to validations in other languages.

Our results revealed a high prevalence of perceived physical fatigability (53.7%) compared to mental fatigability (27.3%) among participants, consistent with previous studies reporting higher rates of physical fatigability in older adults. The elevated prevalence of physical fatigability observed in this study aligns with evidence showing that higher BMI, hip circumference, and visceral fat are associated with greater physical fatigability in individuals aged 65 years and older (38). This finding is significant as it highlights the importance of assessing fatigability to identify individuals at risk of functional decline. Fatigability is prevalent among older adults and is often linked to deteriorating physical function, disability, frailty, and increased mortality. The PFS offers a practical and valid tool for clinicians to measure perceived fatigability, enabling them to develop targeted intervention strategies aimed at enhancing physical and mental health outcomes. By addressing fatigability, healthcare providers can help promote independence and autonomy, supporting healthier aging (8,39).

One of the strengths of this study is the sample size, which exceeded the minimum recommended for psychometric analysis, enhancing the reliability of our findings. Additionally, the study provided a comprehensive evaluation of the PFS-Brasil's psychometric properties, including reliability, validity, and convergent validity. Furthermore, we followed a rigorous methodology for cultural adaptation, adhering to established guidelines, ensuring the scale's relevance and applicability in the Brazilian context (13). The limitations of this study include its cross-sectional design, which prevented the establishment of causal inferences, and the sample composition, which featured a higher prevalence of functionally and cognitively healthy female participants. Additionally, the demographic homogeneity of the sample presented a limitation. However, this aspect warrants further exploration, as the population from the study region reflected the genetic diversity and miscegenation typical of the Brazilian population, making it representative of the country's multiracial characteristics (40). Future studies should investigate the use of the PFS-Brasil in more diverse populations, including individuals with varying health conditions. Moreover, research exploring the scale's sensitivity to changes resulting from interventions targeting fatigability would provide valuable insights into its effectiveness in monitoring improvements over time.

## Conclusion

The PFS-Brasil demonstrated strong psychometric properties, establishing itself as a reliable and valid instrument for assessing perceived physical and mental fatigue in older Brazilian adults. Its ability to accurately reflect fatigability, coupled with its ease of use, makes it a valuable tool for both clinical and research settings. The scale's association with physical and cognitive performance measures highlights its potential for identifying individuals at risk of functional decline, supporting timely interventions to promote healthier aging.

## Supplementary Materials

Supplementary Material Click to view [pdf].

## Data Availability

The datasets generated and/or analyzed during the current study are available from the corresponding author on reasonable request.
