# Supplementary material for: Psychometric properties of the Pittsburgh Fatigability Scale for assessing physical and mental fatigability in Brazilian older adults (PFS-Brasil)
Source: Braz J Med Biol Res. 2026 Feb 16;59:e14619. doi: 10.1590/1414-431X2025e14619 (PMC12919755; doi:10.1590/1414-431X2025e14619)
Supplement: Supplementary Material [file 1414-431X-bjmbr-59-e14619-suppl.pdf]

Table S1. Reliability and internal consistency properties of the Pittsburgh Fatigability Scale - Brasil version (PFS-Brasil) (n=103).

PFS-Brasil	Test (mean ± SD)	Retest (mean ± SD)	Floor effect n (%)	Ceiling effect n (%)	Cronbach alpha	ICC (95%CI)	SEM (%)	SDC	SDC (%)
Physical subscale score	15.0 ± 9.3	15.2 ± 9.2	0 (0)	0 (0)	0.80	0.84 (0.80–0.88)	7%	10	20%
Mental subscale score	7.8 ± 7.7	6.9 ± 8.0	29 (24)*	0 (0)	0.78	0.83 (0.78–0.87)	6%	8	17%

SD: standard deviation; ICC: intraclass correlation coefficient for agreement using 2-way mixed effect model; SEM: standard error of measurement; SDC: smallest detectable change; *floor effect.
